# Supplementary material for: CD47 is required for mesenchymal progenitor proliferation and fracture repair
Source: Bone Res. 2025 Mar 3;13:29. doi: 10.1038/s41413-025-00409-0 (PMC11873311; doi:10.1038/s41413-025-00409-0)
Supplement: Supplementary file 1 — Supplemental Figures [file 41413_2025_409_MOESM1_ESM.pdf]

Supplemental Figures

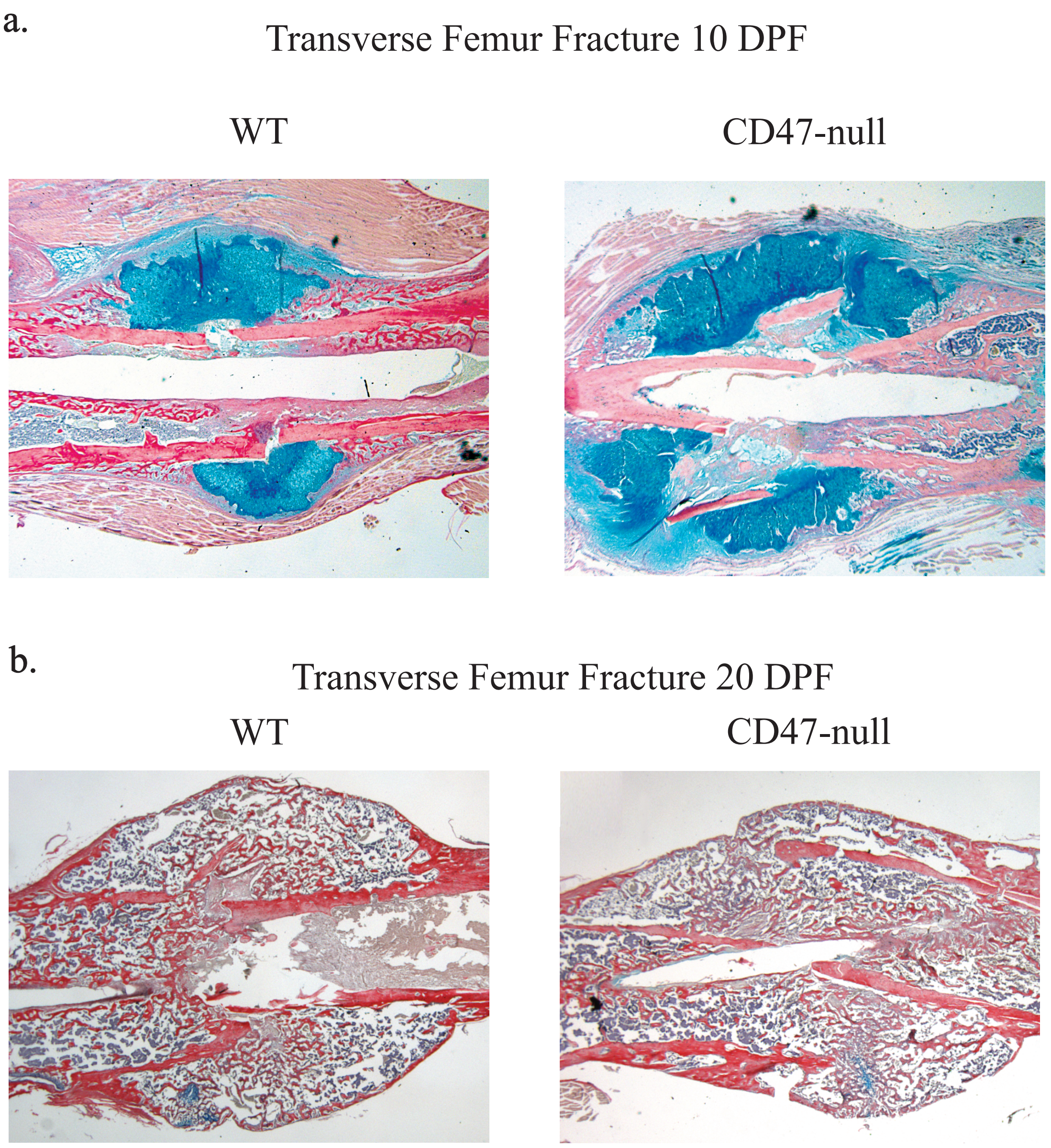

Supplemental Figure 1: Representative histology of transverse femoral fractures. Milligan's Trichrome staining of 10 µm sections of transverse femur fractures in WT (n=6-7) and CD4-null (n=11-13) mice at **a.** 10 and **b.** 20 days post fracture (dpf).

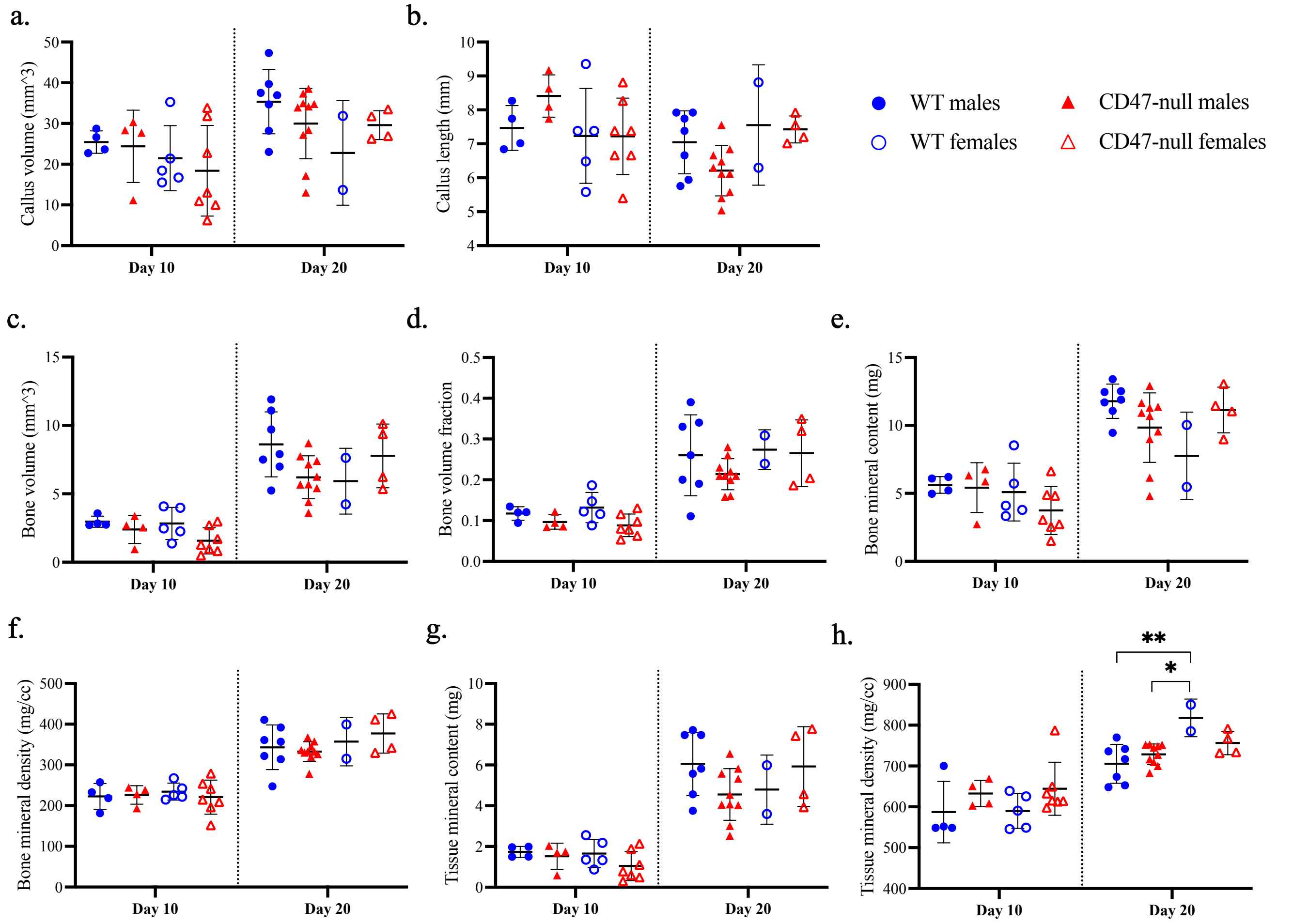

Supplemental Figure 2: Sex-specific analysis of µCT in WT and CD47-null femoral fracture calluses at 10 and 20 days post fracture. a-h, Quantitative assessments of various bone healing parameters determined by µCT (mean±SD) at day 10 and 20 post-fracture in WT (n = 9) and CD47-null (n = 11-14) mice. Each data point represents an individual mouse. **a.** Callus volume, **b.** Callus length, **c.** Bone volume, **d.** Bone volume fraction, **e.** Bone mineral content, **f.** Bone mineral density, **g.** Tissue mineral content, **h.** Tissue mineral density. Closed shapes represent males, open represent females. Data was analyzed via two-way ANOVA, and group comparisons were completed using the Tukey method. *P*<0.05, **P<0.01 indicate significant differences between groups.

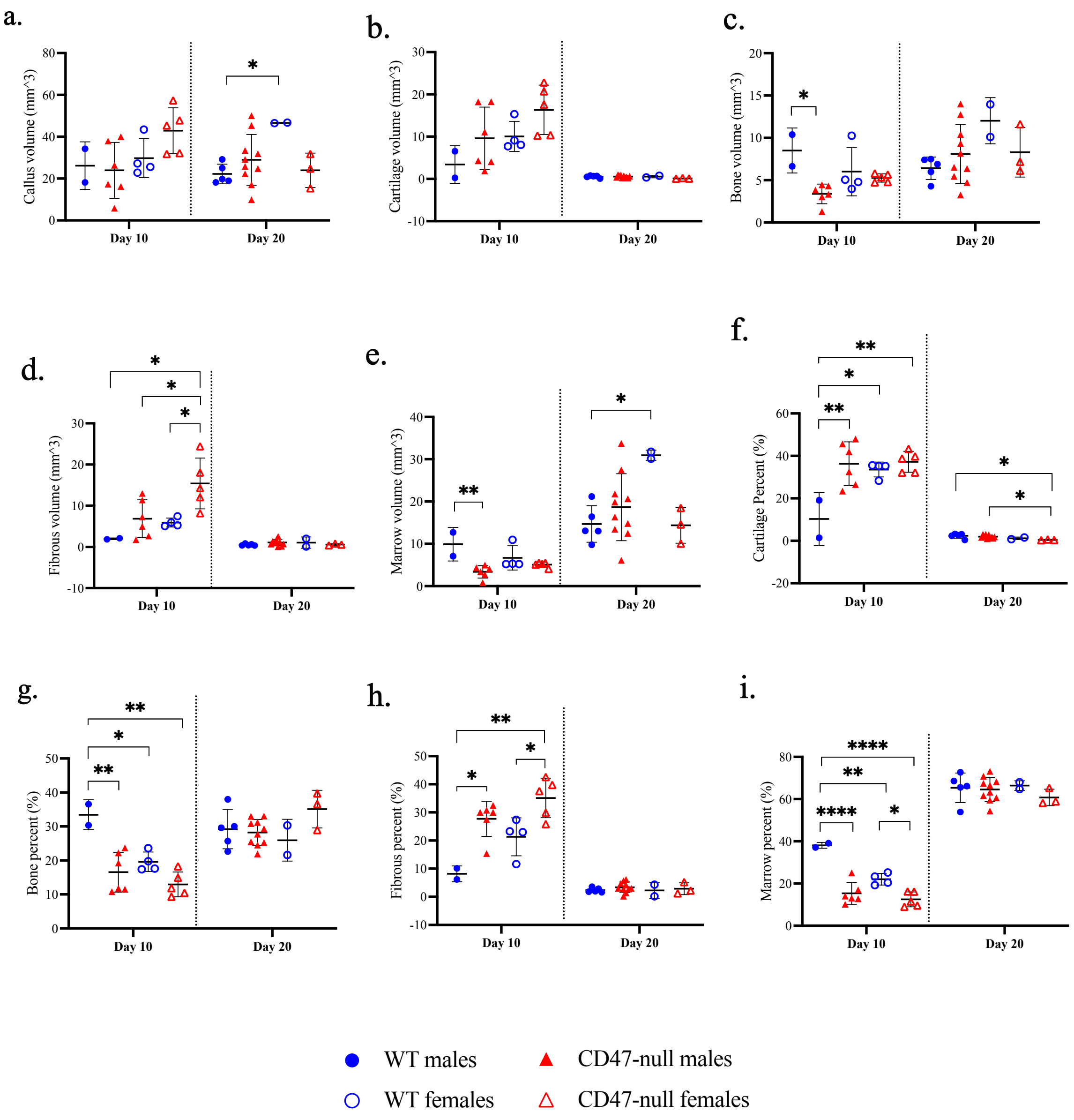

Supplemental Figure 3: Sex-specific analysis of histomorphometric analysis of WT and CD47-null femoral fracture calluses at 10 and 20 days post fracture. a-h, Quantitative assessments of various bone healing parameters determined by histomorphometric analysis (mean±SD) at day 10 and 20 post-fracture in WT (n = 6-7) and CD47-null (n = 11-13). Each data point represents an individual mouse. **a.** Callus volume, **b.** Cartilage volume, **c.** Bone volume, **d.** Fibrous volume, **e.** Marrow volume, **f.** Cartilage Percent (%), **g.** Bone Percent (%), **h.** Fibrous Percent (%), and **i.** Marrow Percent (%). Closed shapes represent males, open represent females. Data was analyzed by two-way ANOVA, and group comparisons were completed using the Tukey method. $P<0.05$, $^{**}P<0.01$, $^{***}P<0.001$, $^{****}P<0.0001$ indicate significant differences between groups.

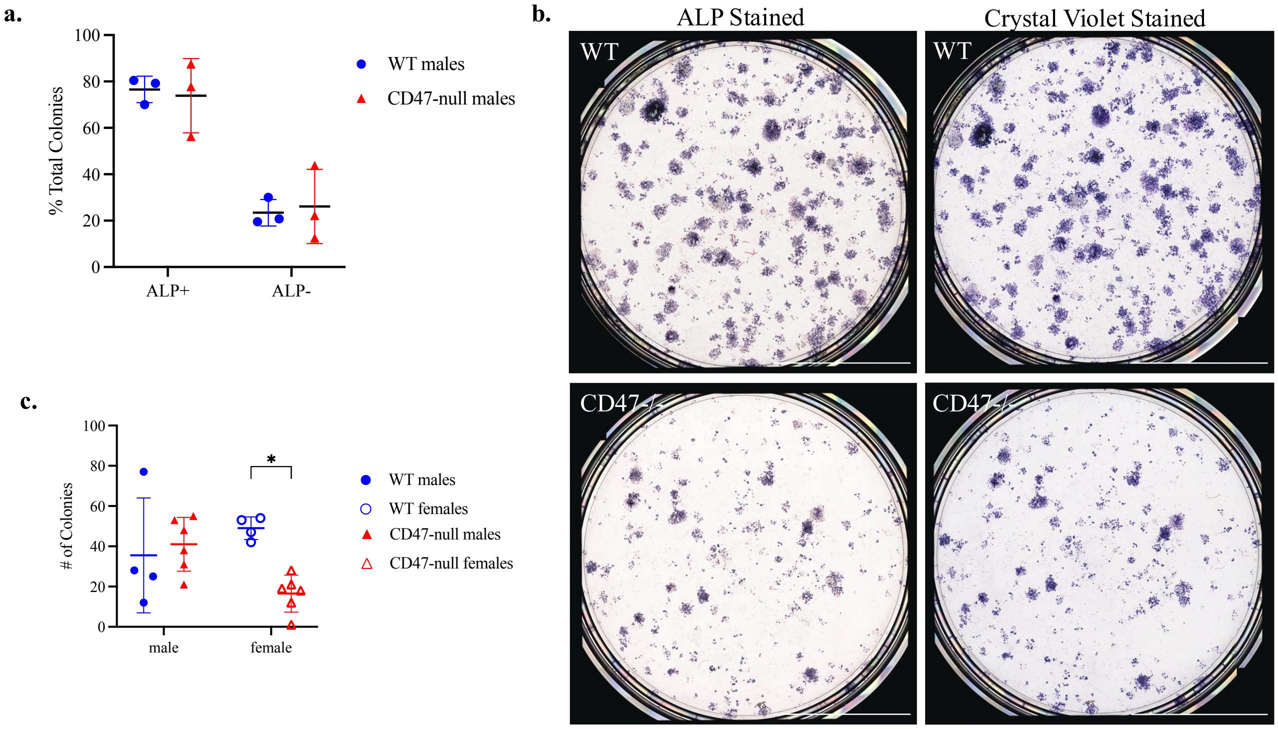

Supplemental Figure 4. Evaluation of CFU colonies in WT and CD47-null genotypes. a. Total ALP+ colonies (large + small) were counted in WT (n = 3) and CD47-null (n = 3) BMSC CFU plates. Total ALP- colonies were also quantified. ALP+ and ALP- colonies were added and the number of ALP+ or ALP- to total colonies was recorded as a proportion for each condition. **b.** Representative images of ALP/Neutral Red stained dishes (left) and Crystal violet-stained dishes (right) in WT (top) and CD47-null (bottom) BMSCs. **c.** Plot of the total number of ALP+ colonies combining large and small colonies from WT (n = 8) and CD47-null (n = 8) mice. Closed shapes represent males, open represent females. Each data point represents an individual mouse. % total colonies **(a)** was analyzed by unpaired t-test. # of colonies **(b)** was analyzed by two-way ANOVA. *P*<0.05, **P<0.01 indicate significant differences between groups.

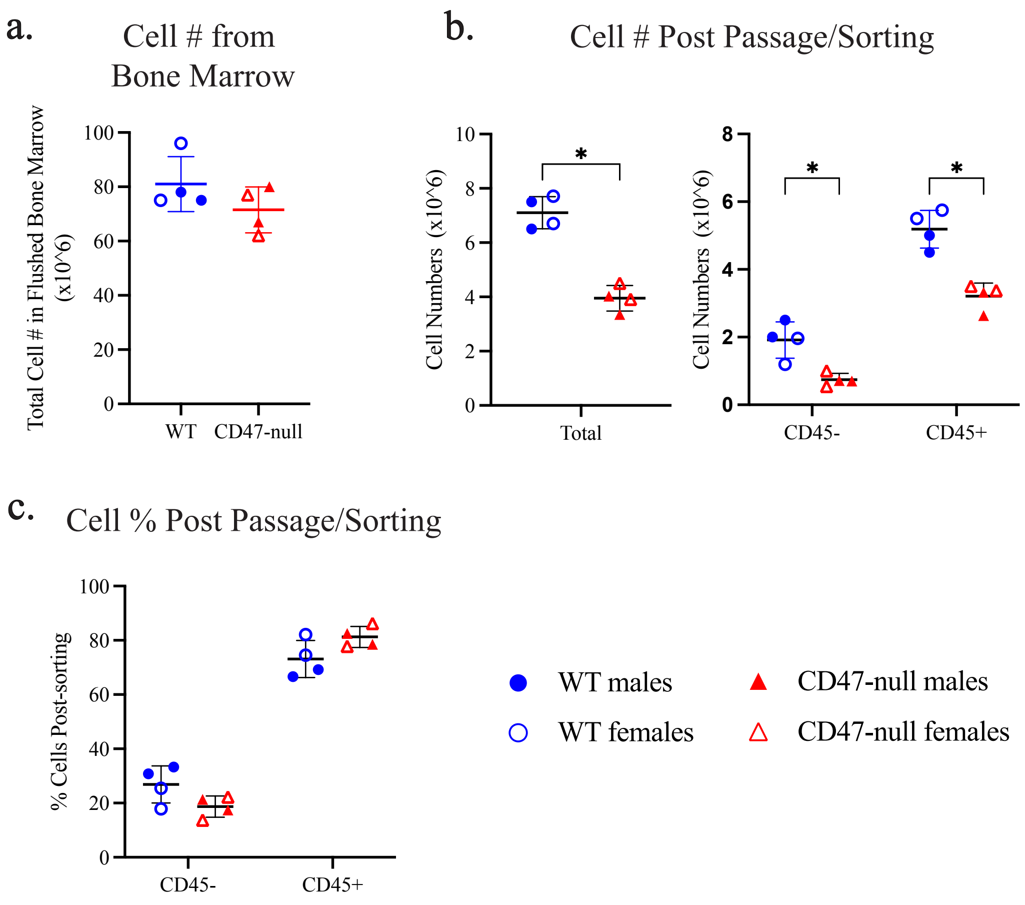

Supplemental Figure 5: Lack of CD47 alters cell proliferation *in vitro*. a. WT (n =4) and CD47-null (n =4) mice have an equivalent initial number of cells from the bone marrow of the femur and tibia. **b.** Bone marrow from CD47-null mice have reduced ability to proliferate (left) in both the stromal (CD45-) and immune population (CD45+) (right). **c.** Bone marrow from WT and CD47-null mice have an equivalent proportion of CD45- to CD45+ cells despite differences in total cell numbers. Each data point represents an individual mouse. *P<0.05, two-sided t-test.

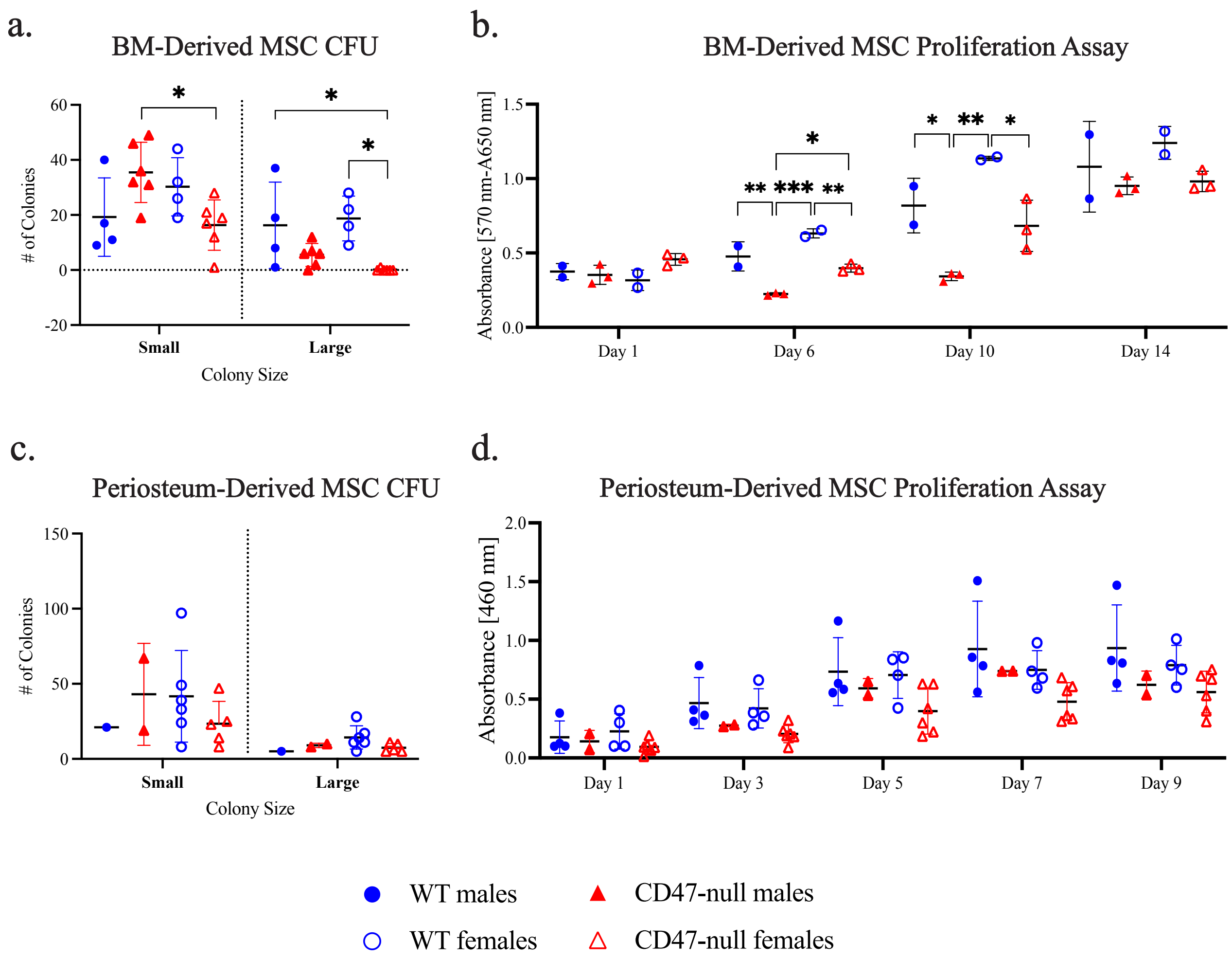

Supplemental Figure 6: Sex-specific analysis of WT and CD47-null MSC proliferation and expansion. a-f, Quantitative assessments of number of colonies formed and cell proliferation of WT (n = 4-8) and CD47-null (n = 6-12) MSC from male and female mice. **a.** CFU-f from primary bone-marrow derived MSC. **b.** MTT assay of primary bone marrow-derived MSC. **c.** CCK8 assay of periosteum-derived MSC. **d.** CFU-f from periosteum-derived MSC. Closed shapes represent males, open represent females. For a, c, and d each data point represents an individual mouse. For b, each data point represents the pooling of cells from 2 mice. Data was analyzed by two-way ANOVA, and group comparisons were completed using the Tukey method. *P*<0.05, **P<0.01, ***P<0.001 indicate significant differences between groups.

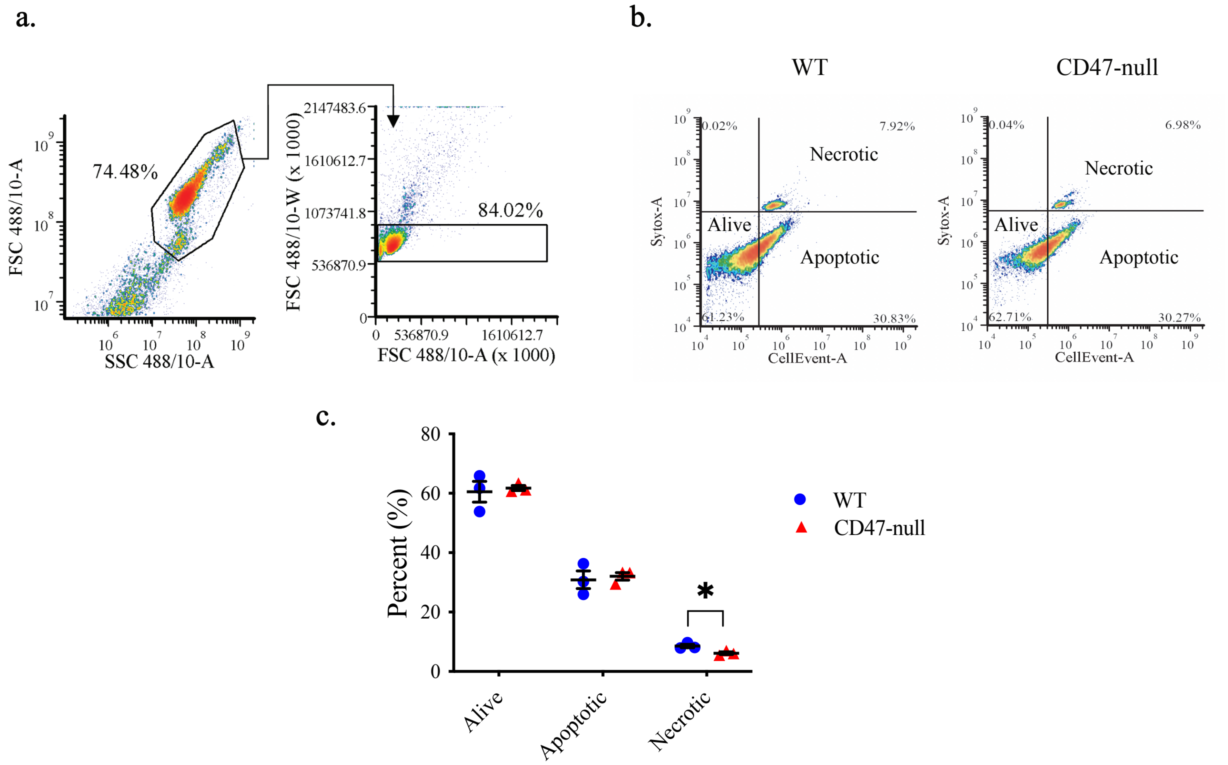

Supplemental Figure 7: Loss of CD47 does not increase mesenchymal stem cell apoptosis. Apoptotic analysis of cells harvested from the femur and tibia of WT (n=3) and CD47-null (n=3) mice. Each data point represents a pooling of 2-3 mice. **a**. Flow cytometry gating scheme for cell density (left) and doublet discrimination (right). **b**. Representative plots of WT and CD47-null with quadrant overlay to segment alive, apoptotic, and apoptotic/necrotic cells. **c**. Comparison of WT and CD47-null population percentage of alive, apoptotic, and apoptotic/necrotic cells. Mean±SD, *$P<0.05$, two-sided t test.

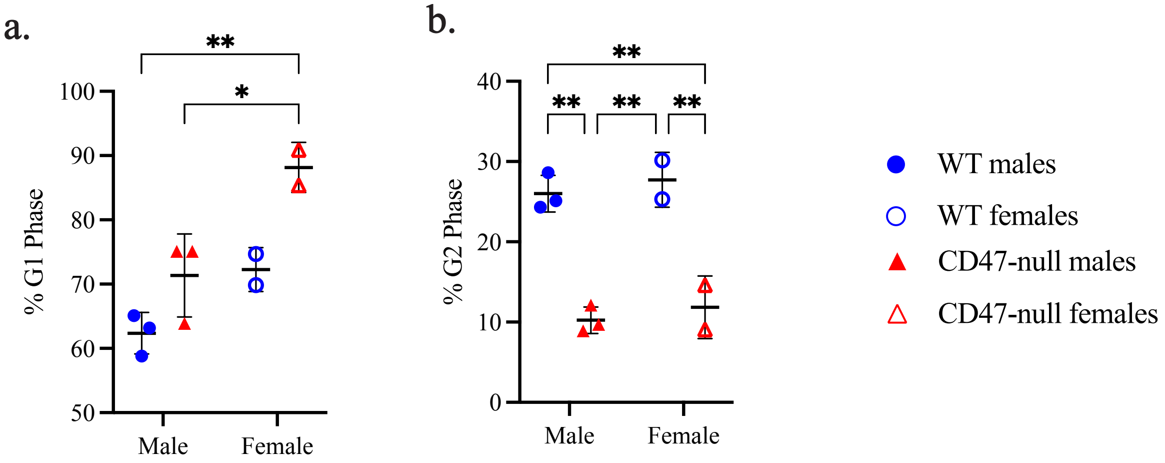

Supplemental Figure 8: Sex-specific analysis of CD47-null and WT MSC in S/G2 Phase. a. Percent of cells in G1 phase from WT (n=5) and CD47-null (n = 5) MSC. Each data point represents a pooling of cells from 2 mice. **b.** Percent of cells in G2 phase. Closed shapes represent males, open represent females. Data was analyzed by two-way ANOVA, and group comparisons were completed using the Tukey method. *P*<0.05, **P<0.01, ***P<0.001 indicate significant differences between groups.

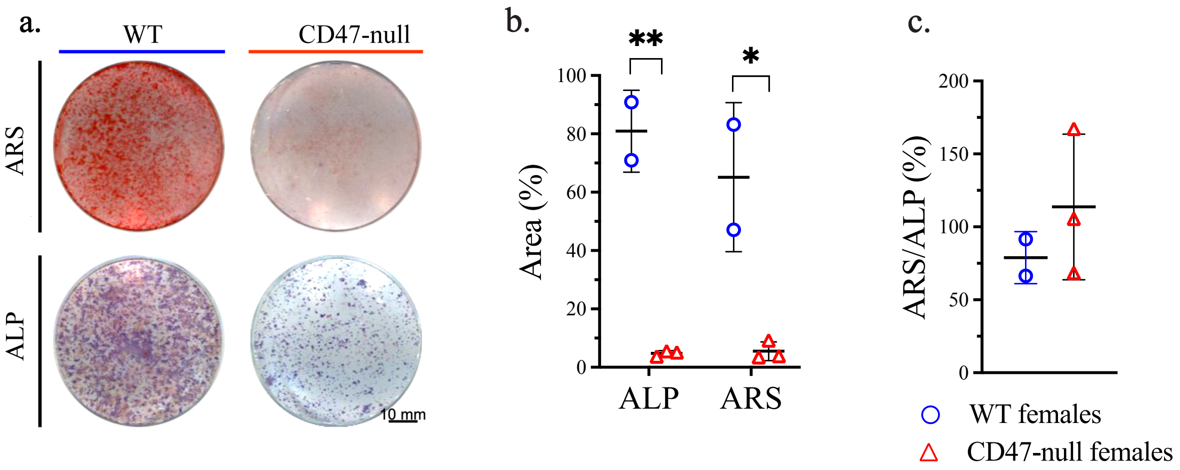

Supplemental Figure 9: Loss of CD47 does not inhibit osteoblast differentiation. Osteogenic analysis of marrow cells harvested from the femur and tibia of WT (n=2) and CD47-null (n=3); each data point represents the pooling of 2 mice. **a**, At day 14, plates with stained with either alizarin red s (ARS) or ALP/Neutral Red. **b,** % Area of ALP and ARS staining. **c,** % area of ARS staining relative to ALP staining. Mean±SD, **P*<0.05, ***P*<0.01, two-sided t test.

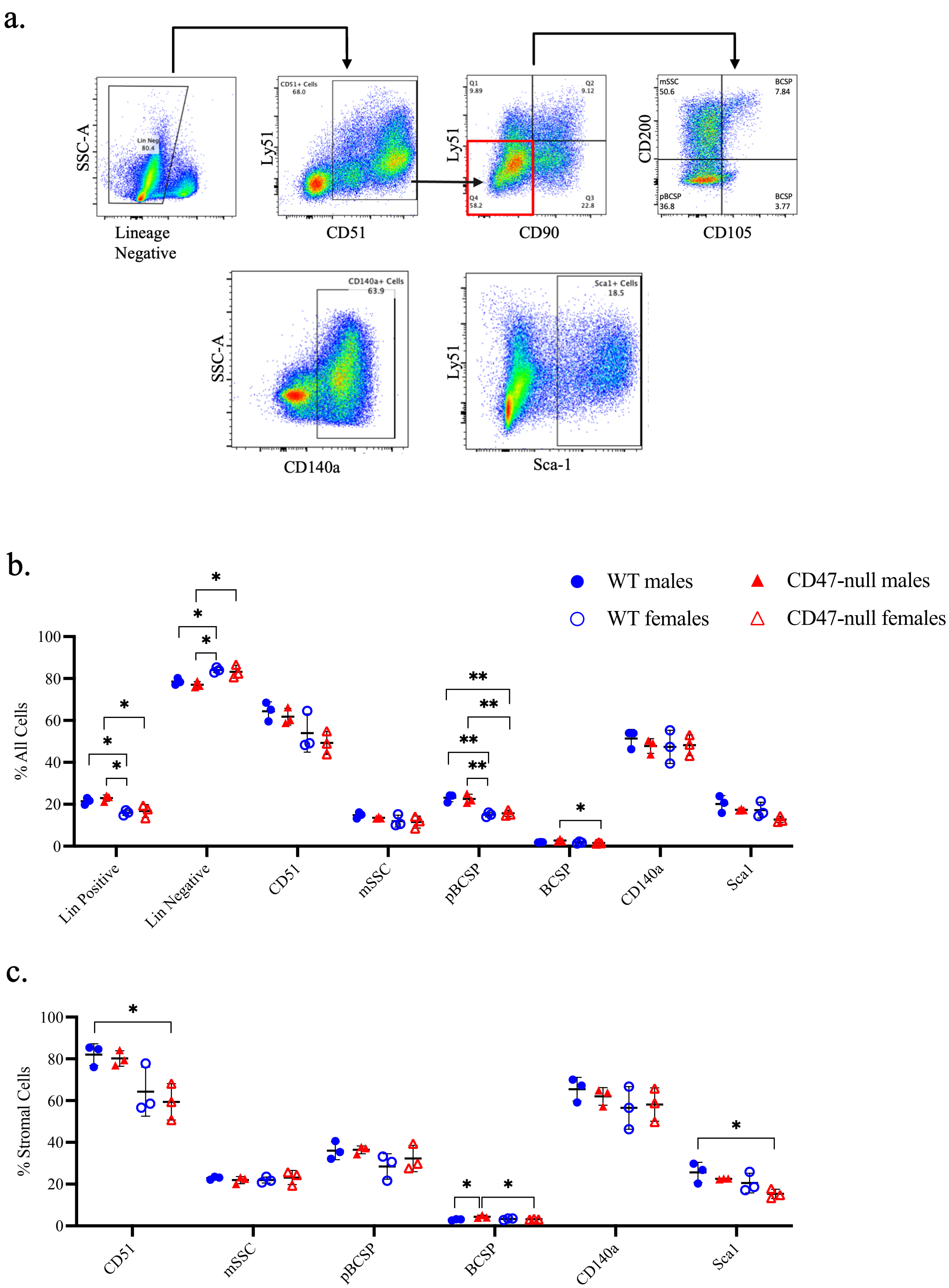

a.
SSC-A
Lin Neg
80.4
Lineage Negative
CD51+ Cells
68.0
Ly51
CD51
Q1
9.89
Q2
9.12
Q4
58.2
Q3
22.8
Ly51
CD90
mSSC
50.6
BCSP
7.84
pBCSP
36.8
BCSP
3.77
CD200
CD105
CD140a+ Cells
63.9
SSC-A
CD140a
Sca1+ Cells
18.5
Ly51
Sca-1
b.
WT males
CD47-null males
WT females
CD47-null females
% All Cells
Lin Positive
Lin Negative
CD51
mSSC
pBCSP
BCSP
CD140a
Sca1
c.
% Stromal Cells
CD51
mSSC
pBCSP
BCSP
CD140a
Sca1

Supplemental Figure 10: Loss of CD47 does not impact baseline skeletal stem cell numbers. Skeletal stem cell marker analysis of periosteal MSC harvested from the femur and tibia of 10-week-old WT (n=6) and CD47-null (n=6) mice Each data point represents an individual mouse. Primary cells were stained with mesenchymal skeletal stem cell panel (fixable viability dye, lineage negative cocktail, CD50, CD90, Ly51, CD105, CD200, CD140a, Sca1), fixed and analyzed for flow cytometry. **a.** Gating scheme for mesenchymal stromal stem cells (mSSC), pre-bone cartilage progenitor cells (pBCSP), bone cartilage progenitor cells (BCSP), CD140a+ cells, and Sca1+ cells. **b.** Plot depicting % of all live cells that each population contributes. **c.** Plot depicting % of total stromal cells (Lineage negative cells) that each population contributes. Closed shapes represent males, open represent females. Data was analyzed by two-way ANOVA, and group comparisons were completed using the Tukey method for each cell population. $P<0.05$, $^{**}P<0.01$ indicate significant differences between groups.

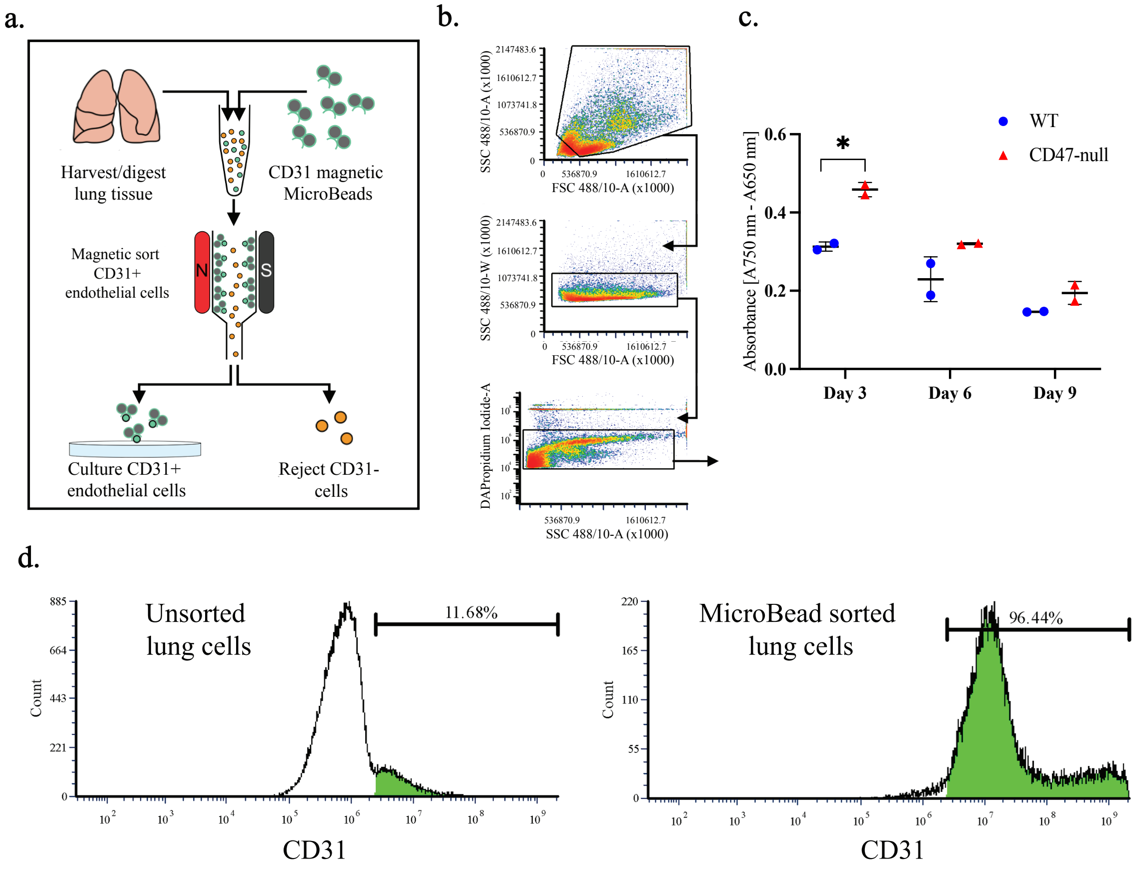

Supplemental Figure 11. Loss of CD47 promotes endothelial cell proliferation

a. Schematic of endothelial cell isolation and culture from the lungs of 4-week-old mice from WT (n =2) and CD47-null mice (n = 2). Each data point represents cells pooled from 4 mice of the same sex. **b**. Flow cytometry gating scheme for lung cell density (top), double discrimination (middle), and alive/dead (bottom). **c**. MTT assay of WT and CD47-null endothelial cells after 3, 6, and 9 days in culture. **d**. Representative histograms of CD31+ lung cells before CD31 MicroBead sorting (left) and after (right). Mean ±SD, **P*<0.05, two-sided t test.

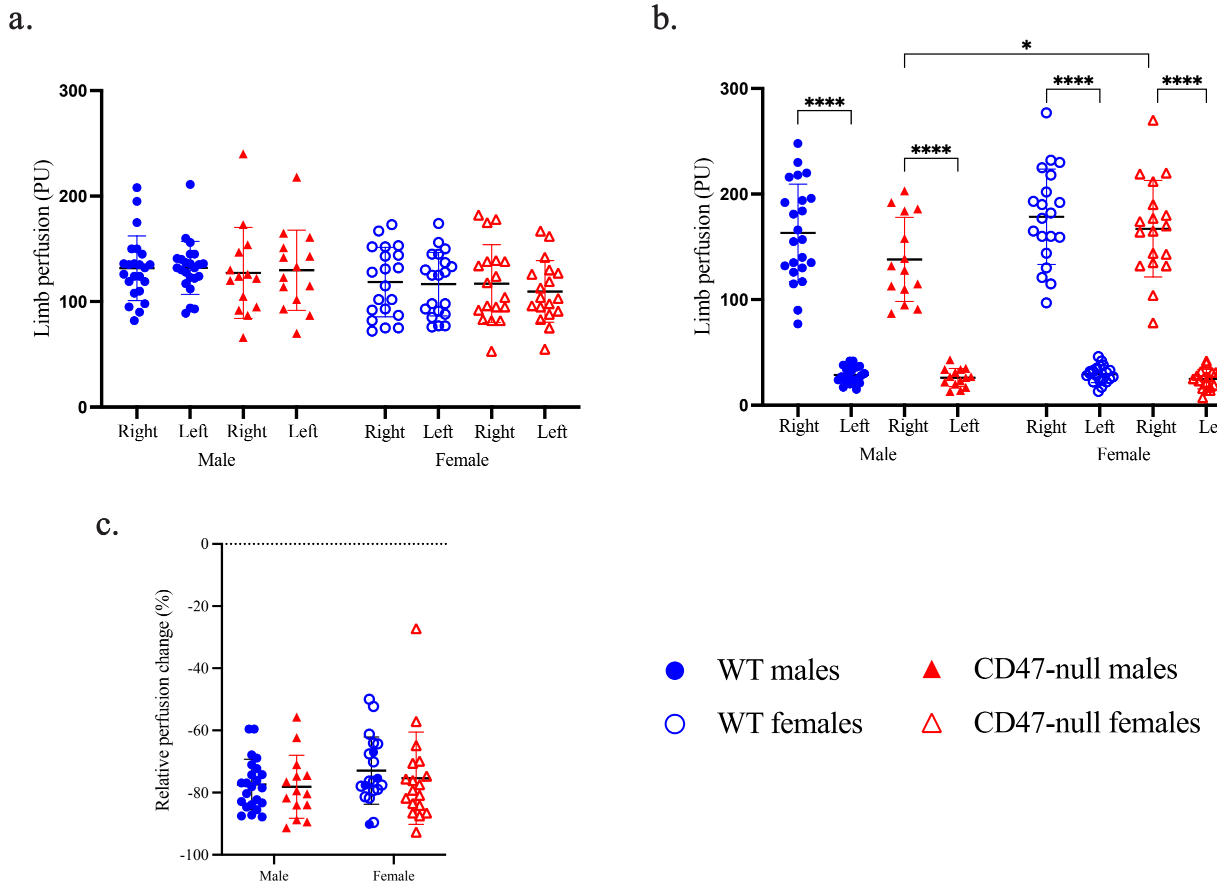

Supplemental Figure 12: Genetic knockout of CD47 has no effect on baseline vascular function or immediate ischemic response. Relative perfusion of WT (n=43) and CD47-null (n=32) mice before ischemia and 9 days post-ischemic tibia fracture using laser doppler flowmetry. Each data point represents an individual mouse. **a**, Left: limb perfusion (mean±SD) pre-ischemic surgery for WT and CD47-null mice in right and left limb. No difference was seen between limb or genotype (one-way ANOVA). Right: limb perfusion (mean±SD) post-ischemic surgery for WT and CD47-null mice in right and left limb (One-way ANOVA). **b,** Relative perfusion change post-ischemia was determined by calculating the percent change in the right limb ratio from pre-ischemia to post-ischemic. WT and CD47-null mice ischemic limb perfusion decreased by -81.83%±1.03 and -82.04%±1.449, respectively. No difference was observed in relative perfusion or between sexes. Closed shapes represent males, open represent females. Data was analyzed by two-way ANOVA, and group comparisons were completed using the Tukey method for each cell population. P<0.05, **P<0.01, ****P<0.0001 indicate significant differences between groups.

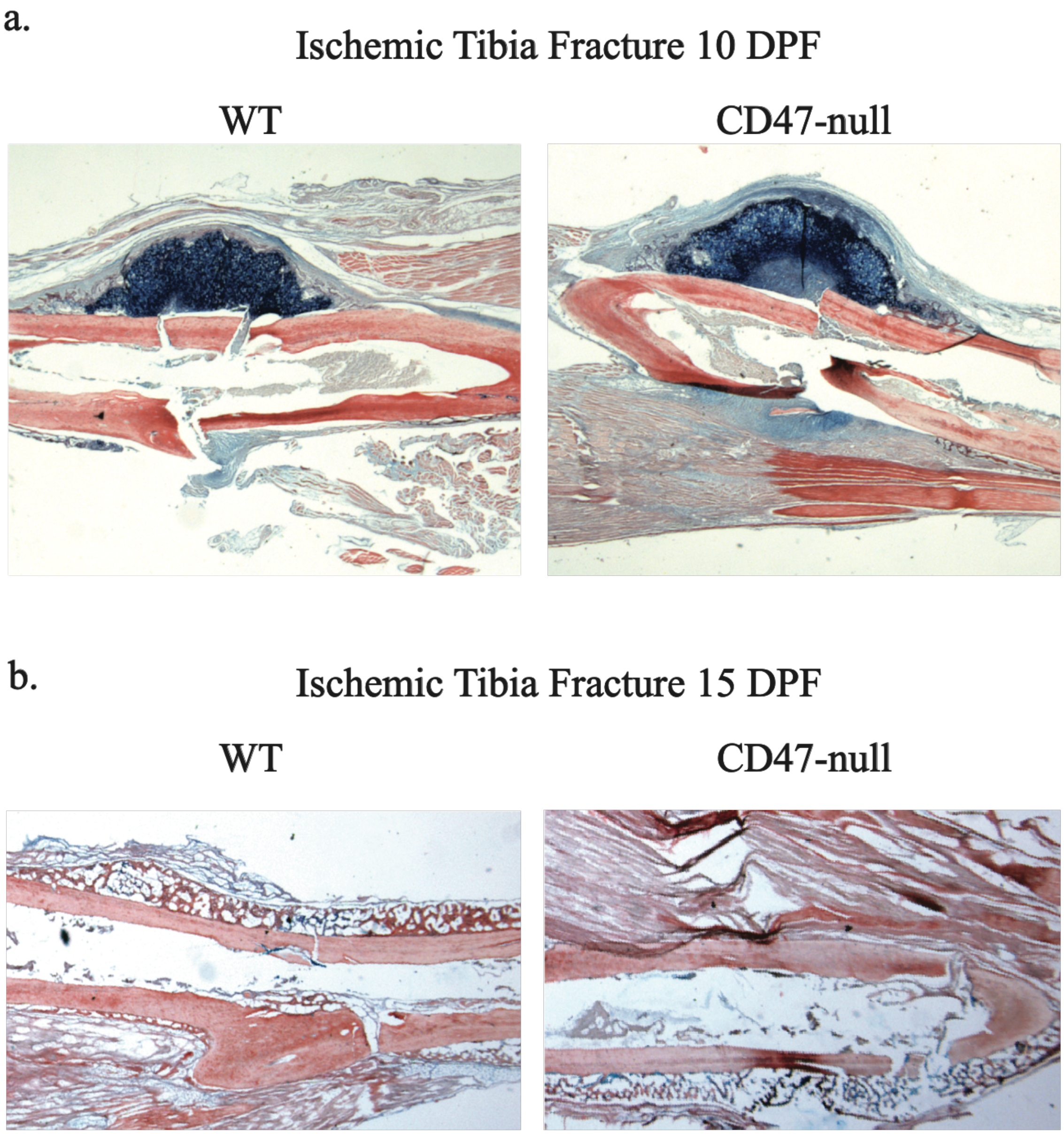

Supplemental Figure 13: Representative histology of transverse ischemic tibia fractures. Milligan's Trichrome staining of 10 μm sections of transverse ischemic tibia fractures in WT (n=8-9) and CD4-null (n=6-8) mice at **a.** 10 and **b.** 15 dpf.

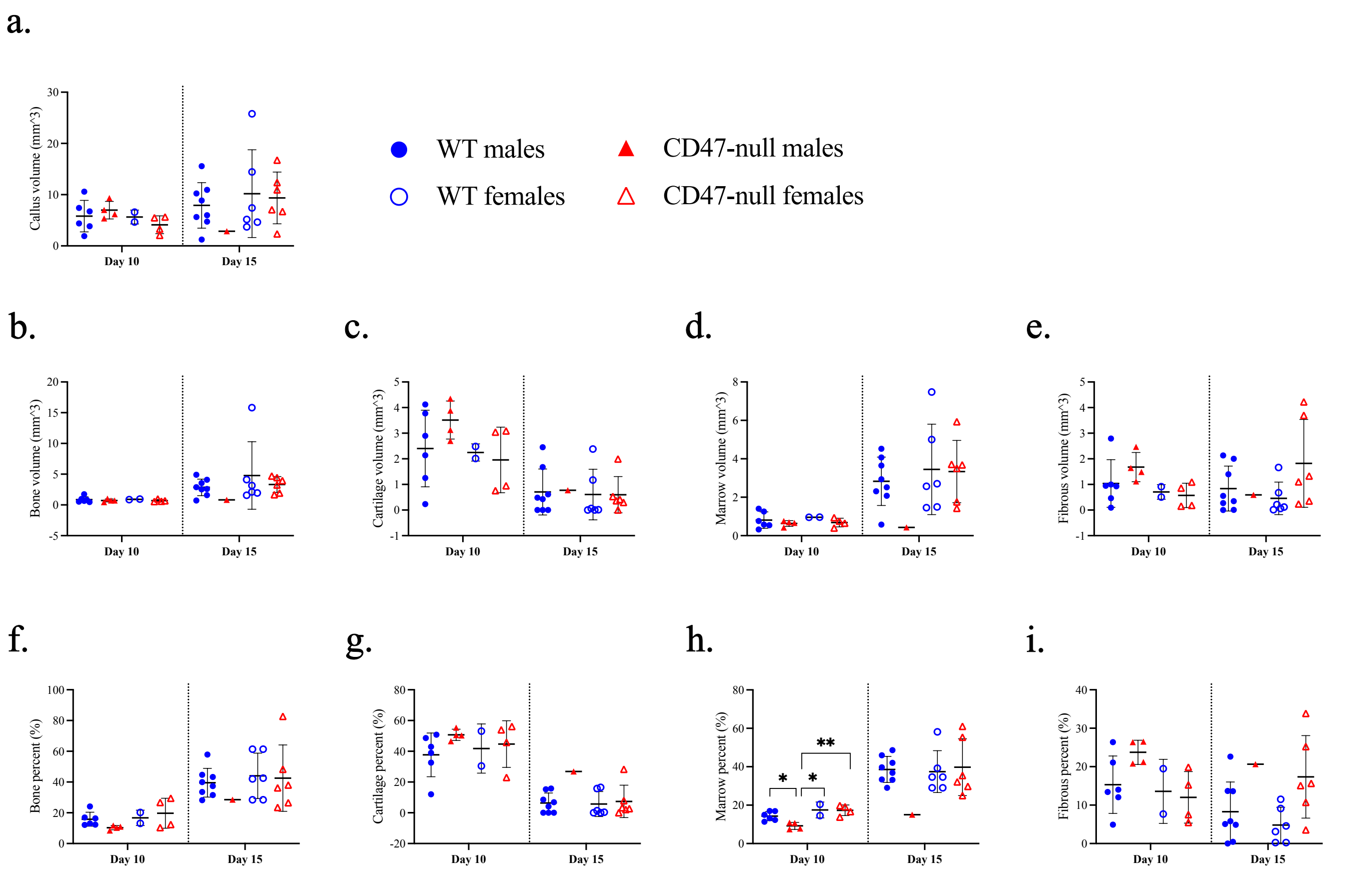

Supplemental Figure 14: Histological data of CD47-null mice with ischemic fracture does not correlate with µCT data. Histomorphometry of ischemic tibia fracture callus of WT (n=8-9) and CD47-null (n=6-8) mice at 10- and 15-days post-fracture. Each data point represents an individual mouse. **a-i**, Callus composition (mean±SD) at day 10 and 15 post-fracture. Data was analyzed by two-way ANOVA, and group comparisons were completed using the Tukey method. *P<0.05, **P<0.01 indicate significant differences between groups.

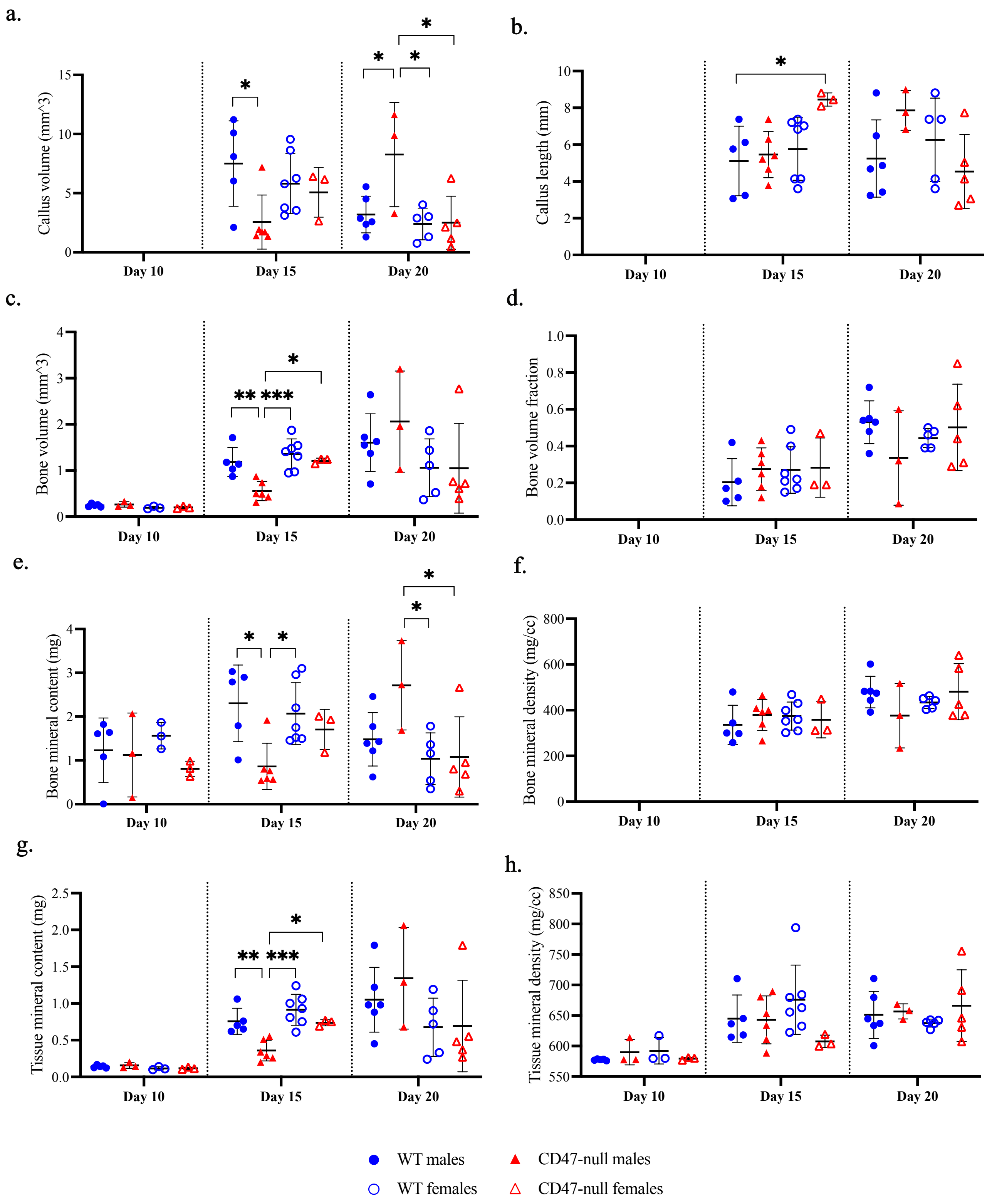

a.
Callus volume (mm^3)
b.
Callus length (mm)
c.
Bone volume (mm^3)
d.
Bone volume fraction
e.
Bone mineral content (mg)
f.
Bone mineral density (mg/cc)
g.
Tissue mineral content (mg)
h.
Tissue mineral density (mg/cc)
Day 10
Day 15
Day 20
WT males
CD47-null males
WT females
CD47-null females

Supplemental Figure 15: Sex-specific analysis of μCT in WT and CD47-null ischemic fracture calluses at 10, 15, and 20 days post fracture. a-h, Quantitative assessments of various bone healing parameters determined by μCT (mean±SD) at day 10, 15, and 20 post-fracture. **a.** Callus volume, **b.** Callus length, **c.** Bone volume, **d.** Bone volume fraction, **e.** Bone mineral content, **f.** Bone mineral density, **g.** Tissue mineral content, **h.** Tissue mineral density. Closed shapes represent males, open represent females. Each data point represents an individual mouse. Data was analyzed by two-way ANOVA, and group comparisons were completed using the Tukey method. *$P<0.05$, **$P<0.01$, ***$P<0.001$ indicate significant differences between groups.

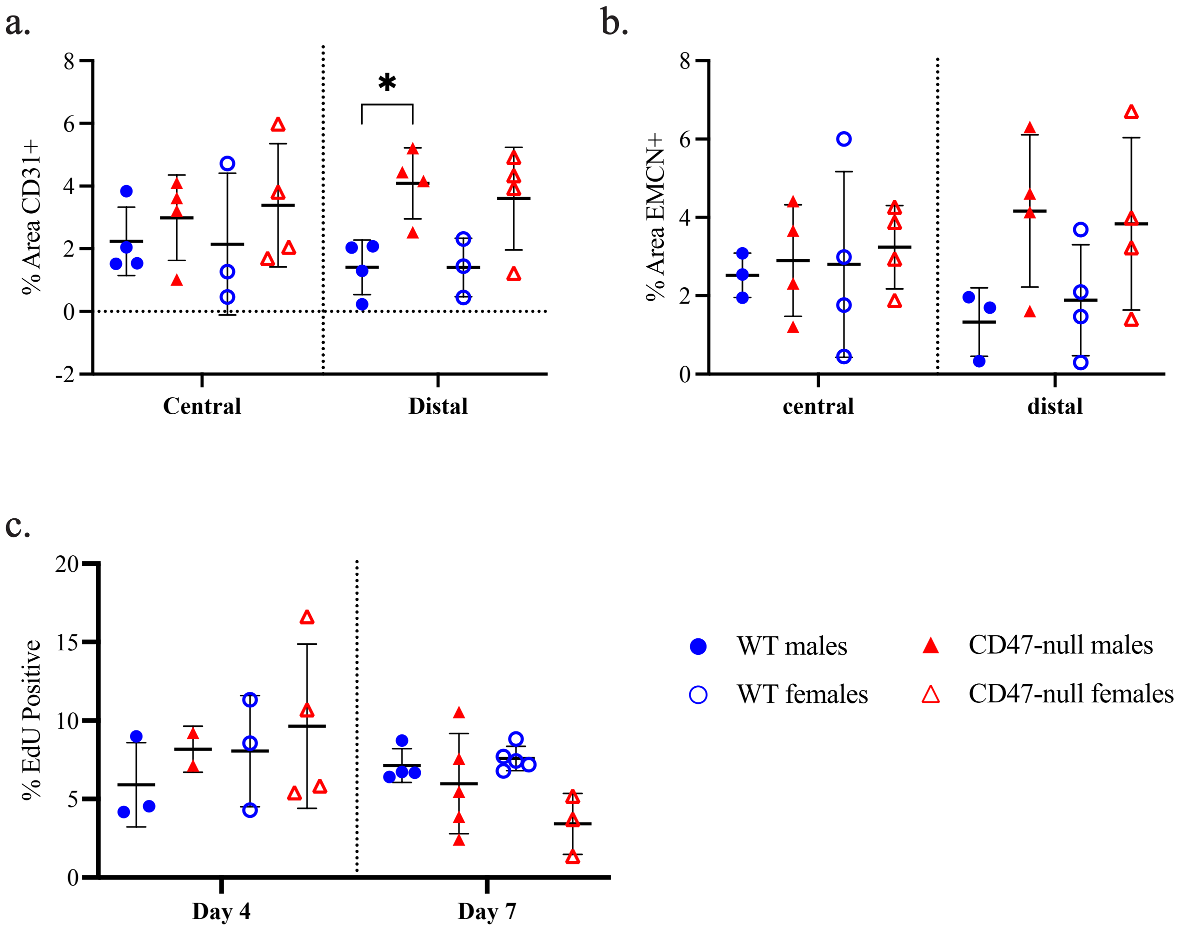

Supplemental Figure 16: Sex-specific analysis of immunofluorescent staining in WT and CD47-null ischemic fracture calluses during early callus formation. a-b, Quantitative assessments of % area of vascular markers (mean±SD) from WT (n = 7) and CD47-null (n = 8) mice. Each data point represents an individual mouse. **a.** CD31, and **b.** EMCN in the central and distal portions of the fracture callus at 4 days post fracture. **c.** Quantitative assessment of % EdU positive nuclei in the fracture callus at 4 and 7 days post fracture. Closed shapes represent males, open represent females. Data was analyzed by two-way ANOVA, and group comparisons were completed using the Tukey method. **P*<0.05 indicates significant differences between groups.

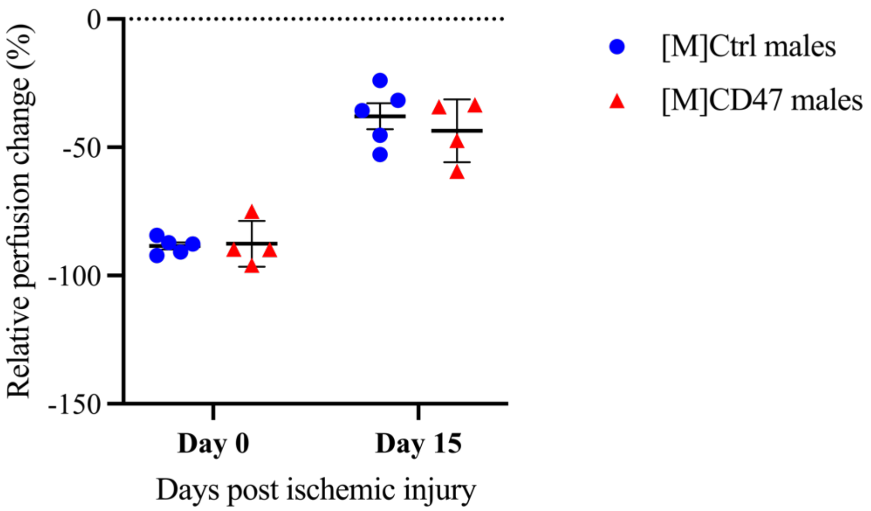

Supplemental Figure 17: Disruption of CD47 using a morpholino does not limit recovery of perfusion after induced ischemia. An ischemic tibial fracture was induced in WT mice prior to intraperitoneal injection with either morpholino-control ([M]Ctrl) (n=5) or morpholino-CD47 ([M]CD47) (n=4) at days 2 and 5 post fracture (1 nmol/kg). Each data point represents an individual mouse. Perfusion measurements were performed prior to ischemia, immediately after, and at day 15 post-ischemia using laser doppler flowmetry of the plantar surface of the hindfoot. Relative perfusion (mean±SD) was calculated for in [M]Ctrl and [M]CD47 mice. One-way ANOVA was performed.

a. Non-Injured Tibia

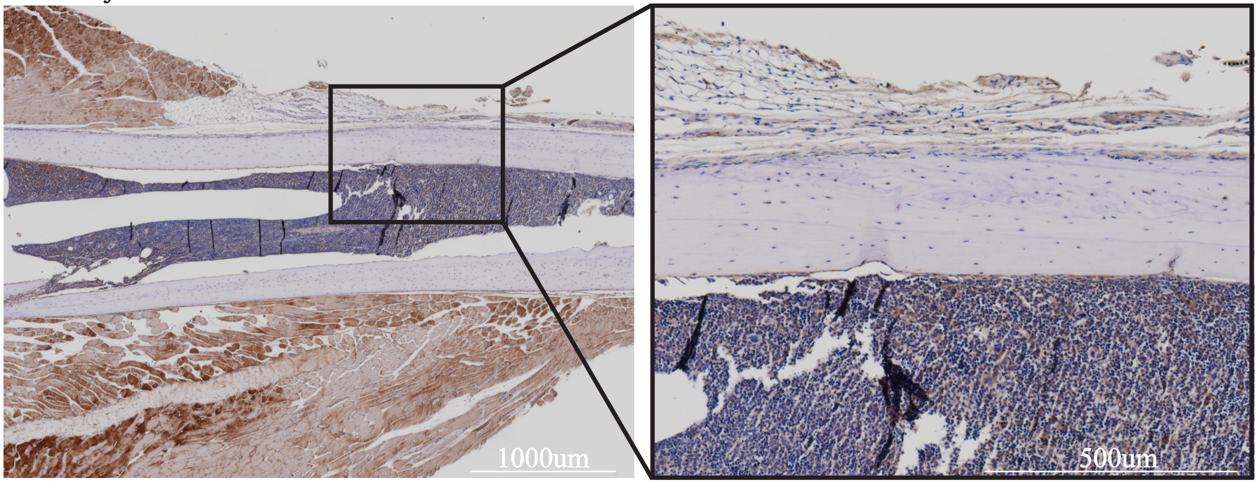

b. 4 Days Post-Fracture

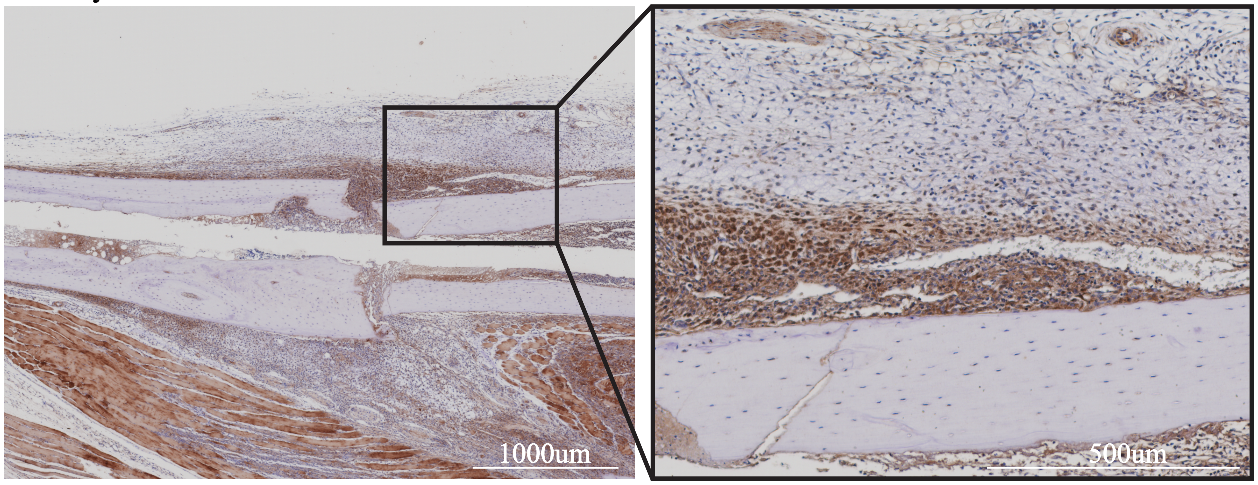

c. 7 Days Post-Fracture

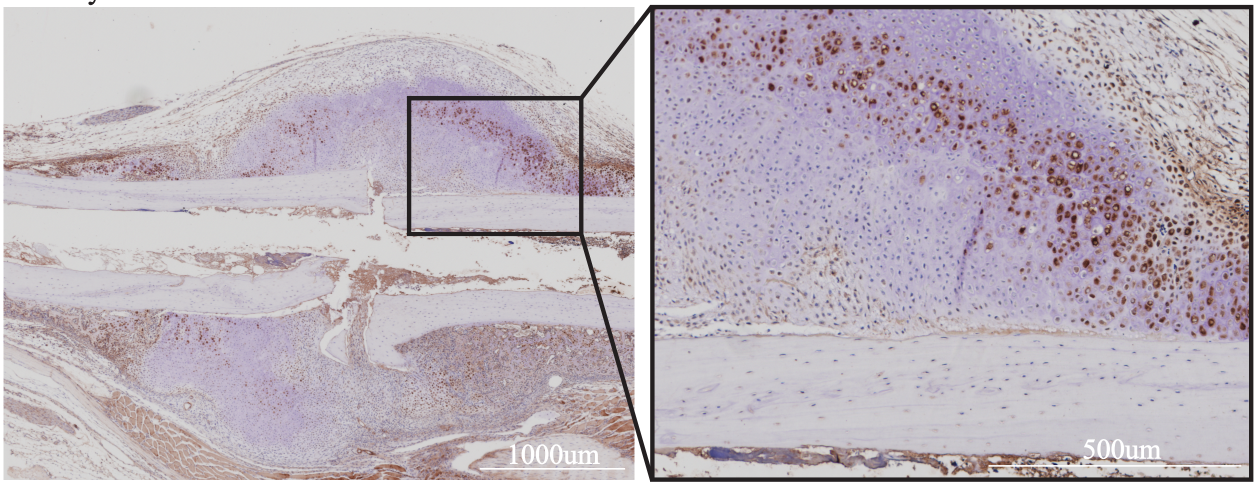

Supplemental Figure 18: CD47 expression in WT healthy tibia and fracture callus. Immunohistochemistry DAB and HRP staining of CD47 in the fracture callus of uninjured tibia and 4 and 7 days post fracture Brown color indicates CD47 positive cells, and blue color indicates hematoxylin counter-stain. Images to the left are 4X stitches of the callus region, with the scale bar indicating 1000 µm. Images to the right are 10X stitched images of a region of the callus representative of various parts of the fracture (bone, callus, bone marrow), with the scale bar indicating 500 µm. **a.** CD47 expression in uninjured tibia. **b.** CD47 expression in the fracture callus at 4 dpf. **c.** CD47 expression in the fracture callus at 7 days post fracture.
