# Supplementary material for: CD47 is required for mesenchymal progenitor proliferation and fracture repair
Source: Bone Res. 2025 Mar 3;13:29. doi: 10.1038/s41413-025-00409-0 (PMC11873311; doi:10.1038/s41413-025-00409-0)
Supplement: Supplementary file 2 — Suppemental Tables [file 41413_2025_409_MOESM2_ESM.docx]

**Supplemental Tables**

| **10 Days Post Femoral Fracture** | | | | | | |
| --- | --- | --- | --- | --- | --- | --- |
| **MicroCT Analysis**  **(2-way ANOVA)** | **Source of variation (p-value)** | | | **% of variation** | | |
| **Parameters** | **Interaction Factor** | **Row Factor (sex)** | **Column Factor (genotype)** | **Interaction Factor** | **Row Factor (sex)** | **Column Factor (genotype)** |
| Callus Length | 0.3411 | 0.1632 | 0.3521 | 4.875 | 10.82 | 4.65 |
| Callus Volume | 0.8052 | 0.2374 | 0.6198 | 0.3482 | 8.342 | 1.417 |
| Bone Volume | 0.4392 | 0.2893 | 0.0527 | 2.663 | 5.083 | 18.52 |
| Bone Mineral Content | 0.4783 | 0.184 | 0.3461 | 2.614 | 9.563 | 4.674 |
| Bone Mineral Density | 0.5736 | 0.8289 | 0.7422 | 1.995 | 0.2917 | 0.6765 |
| Tissue Mineral Content | 0.5015 | 0.3531 | 0.1701 | 2.328 | 4.503 | 10.16 |
| Tissue Mineral Density | 0.8684 | 0.789 | 0.0759 | 0.1412 | 0.3687 | 17.93 |
| Bone Volume Fraction | 0.371 | 0.7994 | 0.0206* | 3.44 | 2712 | 26.78 |
| **20 Days Post Femoral Fracture** | | | | | | |
| **MicroCT Analysis**  **(2-way ANOVA)** | **Source of variation (p-value)** | | | **% of variation** | | |
| **Parameters** | **Interaction Factor** | **Row Factor (sex)** | **Column Factor (genotype)** | **Interaction Factor** | **Row Factor (sex)** | **Column Factor (genotype)** |
| Callus Length | 0.4149 | 0.0559 | 0.2682 | 2.468 | 14.74 | 4.622 |
| Callus Volume | 0.1465 | 0.1248 | 0.8585 | 9.809 | 11.04 | 0.1397 |
| Bone Volume | 0.0581 | 0.584 | 0.8031 | 16.94 | 1.285 | 0.2647 |
| Bone Mineral Content | 0.0221* | 0.2156 | 0.5089 | 23.82 | 6.29 | 1.737 |
| Bone Mineral Density | 0.4773 | 0.1817 | 0.8235 | 2.353 | 8.605 | 0.2289 |
| Tissue Mineral Content | 0.0965 | 0.9395 | 0.8106 | 12.7 | 0.02459 | 0.2452 |
| Tissue Mineral Density | 0.0270* | 0.0008*** | 0.2911 | 15.74 | 42.98 | 3.231 |
| Bone Volume Fraction | 0.601 | 0.3654 | 0.4429 | 1.298 | 3.927 | 2.804 |

**Supplemental Table 1: Two-way ANOVA analysis of micro-computed tomography of femoral fracture calluses at 10 and 20 days post fracture (Figure 1 and Supplemental Figure 2).** The effect of sex and genotype were assessed in WT and CD47-null fracture calluses via two-way ANOVA. The source of variation and % variation due to sex, genotype, and their interaction is plotted. Significant p-values (< 0.05) are highlighted.

| **10 Days Post Femoral Fracture** | | | | | | |
| --- | --- | --- | --- | --- | --- | --- |
| **Histo Analysis**  **(2-way ANOVA)** | **Source of variation (p-value)** | | | **% of variation** | | |
| **Parameters** | **Interaction Factor** | **Row Factor (sex)** | **Column Factor (genotype)** | **Interaction Factor** | **Row Factor (sex)** | **Column Factor (genotype)** |
| Callus Volume | 0.2332 | 0.09 | 0.3918 | 7.511 | 16.12 | 3.77 |
| Cartilage Volume | 0.992 | 0.0544 | 0.0688 | 0.0005042 | 21.65 | 19.06 |
| Bone Volume | 0.0344* | 0.746 | 0.0070** | 20.46 | 0.3959 | 36.95 |
| Marrow Volume | 0.0376* | 0.4855 | 0.0021** | 17.38 | 1.672 | 47.43 |
| Fibrous Volume | 0.3477 | 0.0204* | 0.0097** | 3.039 | 22.33 | 29.36 |
| Cartilage % | 0.0194* | 0.0129* | 0.0037** | 22.36 | 26.1 | 39.17 |
| Bone % | 0.0523 | 0.0030** | 0.0003*** | 10.52 | 30.44 | 55.42 |
| Marrow % | 0.0083** | 0.0006*** | <0.0001**** | 12.31 | 25.31 | 73.75 |
| Fibrous % | 0.4136 | 0.0097** | 0.0003*** | 1.739 | 22.36 | 58.78 |
| **20 Days Post Femoral Fracture** | | | | | | |
| **Histo Analysis**  **(2-way ANOVA)** | **Source of variation (p-value)** | | | **% of variation** | | |
| **Parameters** | **Interaction Factor** | **Row Factor (sex)** | **Column Factor (genotype)** | **Interaction Factor** | **Row Factor (sex)** | **Column Factor (genotype)** |
| Callus Volume | 0.0126* | 0.0822 | 0.1481 | 30.99 | 13.49 | 9.057 |
| Cartilage Volume | 0.126 | 0.1398 | 0.1791 | 11.36 | 10.52 | 8.606 |
| Bone Volume | 0.1072 | 0.0858 | 0.53 | 13.82 | 15.9 | 1.955 |
| Marrow Volume | 0.009** | 0.1034 | 0.0873 | 33.64 | 11.37 | 12.64 |
| Fibrous Volume | 0.1748 | 0.977 | 0.9105 | 10.65 | 0.004524 | 0.06887 |
| Cartilage % | 0.6855 | 0.0061** | 0.2121 | 0.5974 | 34.97 | 5.931 |
| Bone % | 0.0644 | 0.4851 | 0.1275 | 18.02 | 2.332 | 11.8 |
| Marrow % | 0.4439 | 0.6594 | 0.3085 | 3.504 | 1.147 | 6.293 |
| Fibrous % | 0.8465 | 0.7442 | 0.4013 | 0.2224 | 0.633 | 4.268 |

**Supplemental Table 2: 2-way ANOVA analysis of histomorphometric quantification of femoral fracture calluses at 10 and 20 days post fracture (Figure 2 and Supplemental Figure 3).** The effect of sex and genotype were assessed in WT and CD47-null fracture calluses via two-way ANOVA. The source of variation and % variation due to sex, genotype, and their interaction is plotted. Significant p-values (< 0.05) are highlighted.

| **Characterization of Stromal Cell Proliferation and Colony Formation** | | | | | | |
| --- | --- | --- | --- | --- | --- | --- |
| **Marrow-Derived CFU Analysis** | **Source of variation (p-value)** | | | **% of variation** | | |
|  | **Interaction Factor** | **Row Factor (sex)** | **Column Factor (genotype)** | **Interaction Factor** | **Row Factor (sex)** | **Column Factor (genotype)** |
| Large | 0.2998 | 0.7034 | 0.0010** | 3.415 | 0.4468 | 47.89 |
| Small | 0.0088** | 0.431 | 0.8204 | 32.96 | 2.416 | 0.1972 |
| (Small + Large) | 0.0164* | 0.4489 | 0.0748 | 25.13 | 2.106 | 12.69 |
|  |  |  |  |  |  |  |
| **Marrow-Derived MSC Proliferation** | **Source of variation (p-value)** | | | **% of variation** | | |
|  | **Interaction Factor** | **Row Factor (sex)** | **Column Factor (genotype)** | **Interaction Factor** | **Row Factor (sex)** | **Column Factor (genotype)** |
| Day 1 | 0.0663 | 0.55 | 0.1511 | 33.58 | 2.683 | 18.11 |
| Day 6 | 0.7561 | 0.0013** | 0.0002*** | 0.09598 | 29.07 | 63.48 |
| Day 10 | 0.8966 | 0.0068** | 0.0012** | 0.03281 | 29.22 | 58.41 |
| Day 14 | 0.503 | 0.3421 | 0.0798 | 4.312 | 9.042 | 37.7 |
|  |  |  |  |  |  |  |
| **CD45+/- Cell Sorting** | **Source of variation (p-value)** | | | **% of variation** | | |
|  | **Interaction Factor** | **Row Factor (sex)** | **Column Factor (genotype)** | **Interaction Factor** | **Row Factor (sex)** | **Column Factor (genotype)** |
| Cell # from bone marrow | 0.4214 | 0.748 | 0.261 | 12.08 | 1.787 | 25.8 |
| # CD45- Cells Post Passage/Sorting | 0.2294 | 0.229 | 0.0101* | 7.039 | 4.983 | 73.96 |
| # CD45+ Cells Post Passage/Sorting | 0.4045 | 0.0411* | 0.0009*** | 0.9465 | 9.641 | 85.05 |
| % Cells CD45- | 0.2228 | 0.1267 | 0.0568 | 12.36 | 22.01 | 41.86 |
| % Cells CD45+ | 0.2208 | 0.1261 | 0.0571 | 12.48 | 22.09 | 41.67 |
|  |  |  |  |  |  |  |
| **CD45- MSC Proliferation** | **Source of variation (p-value)** | | | **% of variation** | | |
|  | **Interaction Factor** | **Row Factor (sex)** | **Column Factor (genotype)** | **Interaction Factor** | **Row Factor (sex)** | **Column Factor (genotype)** |
| Day 1 | 0.5172 | 0.1609 | 0.7183 | 6.617 | 38.82 | 1.972 |
| Day 3 | 0.7567 | 0.2653 | 0.2383 | 1.429 | 21.74 | 24.9 |
| Day 5 | 0.6653 | 0.0759 | 0.0602 | 1.307 | 34.09 | 40.56 |
| Day 7 | 0.5515 | 0.1094 | 0.0348* | 2.279 | 22.76 | 53.34 |
| Day 9 | 0.5679 | 0.2196 | 0.0348* | 2.361 | 12.92 | 60.27 |
|  |  |  |  |  |  |  |
| **CD45+ Immune Proliferation** | **Source of variation (p-value)** | | | **% of variation** | | |
|  | **Interaction Factor** | **Row Factor (sex)** | **Column Factor (genotype)** | **Interaction Factor** | **Row Factor (sex)** | **Column Factor (genotype)** |
| Day 1 | 0.5068 | 0.2097 | 0.8061 | 7.769 | 32.65 | 1.007 |
| Day 3 | 0.7858 | 0.6644 | 0.4366 | 1.673 | 4.33 | 14.77 |
| Day 5 | 0.6109 | 0.3904 | 0.5906 | 5.451 | 16.62 | 6.121 |
| Day 7 | 0.146 | 0.2635 | 0.7292 | 35.77 | 18.63 | 1.521 |
| Day 9 | 0.912 | 0.1301 | 0.6231 | 0.1749 | 45.68 | 3.573 |

**Supplemental Table 3: 2-way ANOVA analysis of bone-marrow derived cell proliferation and colony formation (Figure 3a-d and Supplemental Figures 4c, 5, 6a-b).** Analysis was performed on data Marrow-derived MSC CFU-F (corresponds to Figure 3a,b and Supplemental Figure 4c and 6a), Marrow-derived MSC Proliferation (corresponds to Figure 3c and Supplemental Figure 6b), Cell counts post CD45+/- Sorting (corresponds to Supplemental Figure 5), CD45-MSC proliferation by CCK8 (corresponds to Figure 3c), and CD45+ immune cell proliferation by CCK8 (Figure 3d) . The effect of sex and genotype were assessed in WT and CD47-null bone-marrow derived MSC via two-way ANOVA. The source of variation and % variation due to sex, genotype, and their interaction is plotted. Significant p-values (< 0.05) are highlighted.

| **Characterization of Periosteum-derived MSC Proliferation and Colony Formation** | | | | | | | | | | |
| --- | --- | --- | --- | --- | --- | --- | --- | --- | --- | --- |
| **Periosteum-derived MSC Proliferation** | **Source of variation (p-value)** | | | | | **% of variation** | | | | |
|  | **Interaction Factor** | **Row Factor (sex)** | | **Column Factor (genotype)** | | **Interaction Factor** | **Row Factor (sex)** | | **Column Factor (genotype)** | |
| Day 1 | 0.445 | 0.9771 | | 0.1904 | | 4.011 | 0.005542 | | 12.39 | |
| Day 3 | 0.8725 | 0.4687 | | 0.0229* | | 0.1224 | 2.552 | | 30.99 | |
| Day 5 | 0.498 | 0.361 | | 0.0812 | | 2.507 | 4.629 | | 18.6 | |
| Day 7 | 0.7577 | 0.1207 | | 0.1076 | | 0.483 | 13.53 | | 14.66 | |
| Day 9 | 0.7436 | 0.4316 | | 0.0537 | | 0.5953 | 3.519 | | 24.3 | |
|  |  |  | |  | |  |  | |  | |
| **Periosteum-Derived CFU Analysis** | **Source of variation (p-value)** | | | | | **% of variation** | | | | |
|  | **Interaction Factor** | | **Row Factor (sex)** | | **Column Factor (genotype)** | **Interaction Factor** | | **Row Factor (sex)** | | **Column Factor (genotype)** |
| Large | 0.199 | | 0.3536 | | 0.7198 | 12.57 | | 6.289 | | 0.9048 |
| Small | 0.2807 | | 0.9765 | | 0.9179 | 10.96 | | 0.00769 | | 0.09421 |

**Supplemental Table 4: 2-way ANOVA analysis of periosteum- derived stromal cell proliferation and colony formation (Figure 3g-h and Supplemental Figure 6c-d).** Analysis was performed on CCK8 assays (corresponds to Figure 3g-h and Supplemental Figure 6c and Colony formation assays (corresponds to Figure 3h and supplemental figure 6d). The effect of sex and genotype were assessed in WT and CD47-null periosteum-marrow derived MSC via two-way ANOVA. The source of variation and % variation due to sex, genotype, and their interaction is plotted. Significant p-values (< 0.05) are highlighted.

| Gene Expression | **Source of variation (p-value)** | | | **% of variation** | | |
| --- | --- | --- | --- | --- | --- | --- |
|  | **Interaction Factor** | **Row Factor (sex)** | **Column Factor (genotype)** | **Interaction Factor** | **Row Factor (sex)** | **Column Factor (genotype)** |
| CD47 | 0.0772 | 0.0772 | 0.002*** | 1.15 | 1.149 | 84.39 |
| OCT4 | 0.2136 | 0.5279 | 0.4739 | 34.38 | 7.074 | 9.314 |
| KLF | 0.5464 | 0.3333 | 0.3843 | 7.179 | 20.67 | 16.14 |
| MYC | 0.0316* | 0.0773 | 0.0006*** | 4.907 | 2.354 | 75.41 |
| ALP | 0.3985 | 0.3289 | 0.01939 | 10.33 | 14.49 | 29.82 |
| CAP3 | 0.6655 | 0.5024 | 0.0118* | 0.5895 | 1.493 | 78.46 |
| FOXM1 | 0.3528 | 0.105 | 0.0001*** | 0.1438 | 0.6319 | 90.8 |
| G1 Phase | 0.2979 | 0.0045** | 0.0062** | 3.049 | 45.83 | 39.84 |
| G2 Phase | 0.9738 | 0.3704 | <0.0001**** | 0.001245 | 0.3704 | 89.08 |

**Supplemental Table 5: 2-way ANOVA analysis of BMSC gene expression and cell cycle analysis (Figure 4 and Supplemental Figure 8).** Analysis was performed on sex-separated qPCR results (corresponds to Figure 4a-g and cell cycle analysis (corresponds to Figure 4h-i and Supplemental Figure 8). The effect of sex and genotype were assessed in WT and CD47-null periosteum-marrow derived MSC via two-way ANOVA. The source of variation and % variation due to sex, genotype, and their interaction is plotted. Significant p-values (< 0.05) are highlighted.

| **Periosteum Skeletal Stem Cell Phenotyping (% All Cells)** | | | | | | |
| --- | --- | --- | --- | --- | --- | --- |
| **% of Live Cells** | **Source of variation (p-value)** | | | **% of variation** | | |
|  | **Interaction Factor** | **Row Factor (sex)** | **Column Factor (genotype)** | **Interaction Factor** | **Row Factor (sex)** | **Column Factor (genotype)** |
| Lineage Positive | 0.8028 | 0.009*** | 0.3461 | 0.1906 | 74.07 | 2.866 |
| Lineage Negative | 0.8028 | 0.009*** | 0.3461 | 0.1906 | 74.07 | 2.866 |
| CD51+ | 0.7673 | 0.0118* | 0.3312 | 0.476 | 53.47 | 5.434 |
| mSSC | 0.7453 | 0.0875 | 0.5129 | 0.9141 | 30.61 | 3.79 |
| pBCSP | 0.5605 | <0.0001**** | 0.9498 | 0.5707 | 87.04 | 0.006535 |
| BCSP | 0.0672 | 0.026* | 0.2324 | 20.75 | 34.45 | 7.735 |
| CD140a | 0.5069 | 0.5906 | 0.67 | 5.368 | 3.492 | 2.176 |
| Sca1 | 0.6089 | 0.0498* | 0.0597 | 1.539 | 28.94 | 26.09 |
| **Periosteum Skeletal Stem Cell Phenotyping (% Stromal Cells)** | | | | | | |
| **% of Stromal Cells** | **Source of variation (p-value)** | | | **% of variation** | | |
|  | **Interaction Factor** | **Row Factor (sex)** | **Column Factor (genotype)** | **Interaction Factor** | **Row Factor (sex)** | **Column Factor (genotype)** |
| CD51+ | 0.7476 | 0.0031** | 0.4854 | 0.4251 | 66.88 | 2.049 |
| mSSC | 0.3943 | 0.9132 | 0.9348 | 9.176 | 0.1434 | 0.08065 |
| pBCSP | 0.5689 | 0.0745 | 0.484 | 2.695 | 32.09 | 4.112 |
| BCSP | 0.0216* | 0.1261 | 0.0389* | 32.27 | 11.62 | 24.24 |
| CD140a | 0.5686 | 0.1716 | 0.838 | 3.317 | 21.17 | 0.4186 |
| Sca1 | 0.6184 | 0.0172* | 0.0795 | 1.262 | 42.18 | 18.95 |

**Supplemental Table 6: 2-way ANOVA analysis of mSSC populations in the periosteum- (Supplemental Figure 10).** Analysis was performed on each cell population described in the periosteum through flow cytometry (corresponding to Supplemental Figure 10). The effect of sex and genotype were assessed in WT and CD47-null periosteum-marrow derived MSC via two-way ANOVA. The source of variation and % variation due to sex, genotype, and their interaction is plotted. Significant p-values (< 0.05) are highlighted.

| **Limb Perfusion** | | | | | | | |
| --- | --- | --- | --- | --- | --- | --- | --- |
|  | **Source of variation (p-value)** | | | **% of variation** | | | |
|  | **Interaction Factor** | **Row Factor (sex)** | **Column Factor (genotype or limb)** | **Interaction Factor** | **Row Factor (sex)** | **Column Factor (genotype)** | |
| Pre-perfusion | 0.9398 | 0007** | 0.9032 | 0.266 | 4.838 | | 0.3775 |
| Post-perfusion | 0.1804 | 0.0439* | <0.0001**** | 0.6004 | 0.5011 | | 80.55 |
| Relative Change | 0.7511 | 0.1723 | 0.5486 | 0.1381 | 2.589 | | 0.4948 |

**Supplemental Table 7: 2-way ANOVA analysis of perfusion/reperfusion before and after ischemic injury (Supplemental Figure 12).** Sex-specific analysis was performed prior to injury in the right and left limb, after left limb ischemic injury in the right and left limb, and the relative change in perfusion in the left ischemic limb. (corresponding to Supplemental Figure 12). The effect of sex and genotype were assessed in WT and CD47-null mice via two-way ANOVA. The source of variation and % variation due to sex, genotype, and their interaction is plotted. Significant p-values (< 0.05) are highlighted.

| **10 Days Post Ischemic Fracture** | | | | | | |
| --- | --- | --- | --- | --- | --- | --- |
| **Histo Analysis**  **(2-way ANOVA)** | **Source of variation (p-value)** | | | **% of variation** | | |
| **Parameters** | **Interaction Factor** | **Row Factor (sex)** | **Column Factor (genotype)** | **Interaction Factor** | **Row Factor (sex)** | **Column Factor (genotype)** |
| Callus Volume | 0.3163 | 0.2573 | 0.8917 | 7.31 | 9.456 | 0.1294 |
| Cartilage Volume | 0.3085 | 0.2178 | 0.5447 | 7.225 | 10.8 | 2.482 |
| Bone Volume | 0.8394 | 0.9769 | 0.3597 | 0.3304 | 0.006744 | 6.992 |
| Marrow Volume | 0.7723 | 0.5634 | 0.2018 | 0.6312 | 2.543 | 13.14 |
| Fibrous Volume | 0.3304 | 0.0862 | 0.5242 | 5.914 | 20.08 | 2.474 |
| Cartilage % | 0.4812 | 0.8955 | 0.2748 | 3.635 | 0.1238 | 9.009 |
| Bone % | 0.2063 | 0.01345 | 0.7026 | 10.38 | 14.98 | 0.8893 |
| Marrow % | 0.1004 | 0.0014** | 0.0819 | 8.643 | 46.16 | 9.83 |
| Fibrous % | 0.1831 | 0.0813 | 0.3518 | 10.31 | 18.71 | 4.85 |
| **15 Days Post Ischemic Fracture** | | | | | | |
| **Histo Analysis**  **(2-way ANOVA)** | **Source of variation (p-value)** | | | **% of variation** | | |
| **Parameters** | **Interaction Factor** | **Row Factor (sex)** | **Column Factor (genotype)** | **Interaction Factor** | **Row Factor (sex)** | **Column Factor (genotype)** |
| Callus Volume | 0.5761 | 0.2496 | 0.4366 | 1.759 | 7.689 | 3.435 |
| Cartilage Volume | 0.9471 | 0.8 | 0.9575 | 0.02653 | 0.3875 | 0.01711 |
| Bone Volume | 0.8839 | 0.2629 | 0.373 | 0.1154 | 7.039 | 4.394 |
| Marrow Volume | 0.2941 | 0.1134 | 0.2504 | 5.907 | 14.04 | 7.137 |
| Fibrous Volume | 0.2578 | 0.5457 | 0.4274 | 6.296 | 1.746 | 3.036 |
| Cartilage % | 0.0803 | 0.0628 | 0.0419* | 15.18 | 17.39 | 21.25 |
| Bone % | 0.6159 | 0.3344 | 0.5095 | 1.449 | 5.478 | 2.52 |
| Marrow % | 0.0647 | 0.0895 | 0.1234 | 18.03 | 14.99 | 12.15 |
| Fibrous % | 0.988 | 0.4958 | 0.0203* | 0.0008776 | 1.827 | 24.72 |

**Supplemental Table 8: 2-way ANOVA analysis of histomorphometric quantification of ischemic tibia fracture calluses at 10, 15, and 20 days post fracture (Supplemental Figure 13-14).** The effect of sex and genotype were assessed in WT and CD47-null fracture calluses via two-way ANOVA. The source of variation and % variation due to sex, genotype, and their interaction is plotted. Significant p-values (< 0.05) are highlighted.

| **10 Days Post Ischemic Fracture** | | | | | | |
| --- | --- | --- | --- | --- | --- | --- |
| **MicroCT Analysis**  **(2-way ANOVA)** | **Source of variation (p-value)** | | | **% of variation** | | |
| **10 Days post fracture - ischemic fractures** | **Interaction Factor** | **Row Factor (sex)** | **Column Factor (genotype)** | **Interaction Factor** | **Row Factor (sex)** | **Column Factor (genotype)** |
| Bone Volume | 0.7231 | 0.0203* | 0.5096 | 0.7484 | 42.77 | 2.635 |
| Bone Mineral Content | 0.3859 | 0.9831 | 0.2554 | 6.817 | 0.003889 | 12.07 |
| Tissue Mineral Content | 0.5719 | 0.0342* | 0.5324 | 2.098 | 36.9 | 2.569 |
| Tissue Mineral Density | 0.1194 | 0.7804 | 0.9771 | 22 | 0.6225 | 0.006584 |
| **15 Days Post Ischemic Fracture** | | | | | | |
| **MicroCT Analysis**  **(2-way ANOVA)** | **Source of variation (p-value)** | | | **% of variation** | | |
| **Parameters** | **Interaction Factor** | **Row Factor (sex)** | **Column Factor (genotype)** | **Interaction Factor** | **Row Factor (sex)** | **Column Factor (genotype)** |
| Callus Length | 0.113 | 0.019* | 0.0449* | 10.24 | 24.67 | 17.21 |
| Callus Volume | 0.1104 | 0.7477 | 0.0363* | 10.67 | 0.4022 | 19.44 |
| Bone Volume | 0.0705 | 0.0039** | 0.0063** | 7.719 | 23.08 | 20.14 |
| Bone Mineral Content | 0.1022 | 0.3422 | 0.0101* | 9.298 | 2.974 | 26.07 |
| Bone Mineral Density | 0.3851 | 0.7936 | 0.6956 | 4.375 | 0.389 | 0.8719 |
| Tissue Mineral Content | 0.1739 | 0.0033** | 0.0018** | 3.81 | 22.09 | 22.75 |
| Tissue Mineral Density | 0.1211 | 0.9175 | 0.1014 | 11.9 | 0.04935 | 13.41 |
| Bone Volume Fraction | 0.631 | 0.5367 | 0.4841 | 1.317 | 2.188 | 2.817 |
| **20 Days Post Ischemic Fracture** | | | | | | |
| **MicroCT Analysis**  **(2-way ANOVA)** | **Source of variation (p-value)** | | | **% of variation** | | |
| **Parameters** | **Interaction Factor** | **Row Factor (sex)** | **Column Factor (genotype)** | **Interaction Factor** | **Row Factor (sex)** | **Column Factor (genotype)** |
| Callus Length | 0.0388* | 0.2466 | 0.6506 | 24.67 | 6.999 | 1.029 |
| Callus Volume | 0.0367* | 0.0085** | 0.0305* | 17.37 | 30.31 | 18.86 |
| Bone Volume | 0.5493 | 0.0595 | 0.5663 | 1.949 | 21.6 | 1.786 |
| Bone Mineral Content | 0.1191 | 0.0114* | 0.0981 | 10.53 | 31.99 | 11.98 |
| Bone Mineral Density | 0.104 | 0.5078 | 0.5307 | 16.38 | 2.518 | 2.253 |
| Tissue Mineral Content | 0.5851 | 0.0568 | 0.543 | 1.609 | 22.02 | 2.003 |
| Tissue Mineral Density | 0.5341 | 0.913 | 0.3578 | 2.46 | 0.07505 | 5.467 |
| Bone Volume Fraction | 0.1376 | 0.6252 | 0.4103 | 13.72 | 1.388 | 4.002 |

**Supplemental Table 9: 2-way ANOVA analysis of micro-computed tomography of ischemic tibia fracture calluses at 10, 15, and 20 days post fracture (Figure 5 and Supplemental Figure 15).** The effect of sex and genotype were assessed in WT and CD47-null fracture calluses via two-way ANOVA. The source of variation and % variation due to sex, genotype, and their interaction is plotted. Significant p-values (< 0.05) are highlighted.

| **Endothelial Cell Analysis 4 days post fracture** | | | | | | |
| --- | --- | --- | --- | --- | --- | --- |
| **Endothelial Cell Analysis** | **Source of variation (p-value)** | | | **% of variation** | | |
|  | **Interaction Factor** | **Row Factor (sex)** | **Column Factor (genotype)** | **Interaction Factor** | **Row Factor (sex)** | **Column Factor (genotype)** |
| CD31 Central | 0.7871 | 0.8625 | 0.2784 | 0.6171 | 0.253 | 10.47 |
| CD31 Distal | 0.7099 | 0.7019 | 0.0025** | 0.5465 | 0.5786 | 56.94 |
| Emcn Central | 0.968 | 0.7128 | 0.6274 | 0.01478 | 1.254 | 2.191 |
| Emcn Distal | 0.632 | 0.9015 | 0.0231* | 1.319 | 0.0889 | 38.53 |

**Supplemental Table 10: 2-way ANOVA analysis of immunofluorescent quantification of blood endothelial cells in ischemic tibia fracture calluses at 4 days post fracture (Figure 6 and Supplemental Figure 16a-b).** The effect of sex and genotype on endothelial cell density (CD31+ and Emcn+ cells) was assessed in WT and CD47-null fracture calluses via two-way ANOVA. The source of variation and % variation due to sex, genotype, and their interaction is plotted. Significant p-values (< 0.05) are highlighted.

| **Cell Proliferation 4 and 7 days post fracture** | | | | | | |
| --- | --- | --- | --- | --- | --- | --- |
| **EdU Analysis** | **Source of variation (p-value)** | | | **% of variation** | | |
|  | **Interaction Factor** | **Row Factor (sex)** | **Column Factor (genotype)** | **Interaction Factor** | **Row Factor (sex)** | **Column Factor (genotype)** |
| 4 days post-fracture | 0.8872 | 0.4623 | 0.4338 | 0.2247 | 6.243 | 7.112 |
| 7 days post-fracture | 0.1601 | 0.3155 | 0.0206* | 10.19 | 5.003 | 31.87 |

**Supplemental Table 11: 2-way ANOVA analysis of immunofluorescent quantification of proliferating cells in ischemic tibia fracture calluses at 4 and 7 days post fracture (Figure 7 and Supplemental Figure 16c).** The effect of sex and genotype on cell proliferation via EdU was assessed in WT and CD47-null fracture calluses via two-way ANOVA. The source of variation and % variation due to sex, genotype, and their interaction is plotted. Significant p-values (< 0.05) are highlighted.
